# Vitamin D, Depressive Symptoms, and Covid-19 Pandemic

**DOI:** 10.3389/fnins.2021.670879

**Published:** 2021-05-13

**Authors:** Gilciane Ceolin, Giulia Pipolo Rodrigues Mano, Natália Schmitt Hames, Luciana da Conceição Antunes, Elisa Brietzke, Débora Kurrle Rieger, Júlia Dubois Moreira

**Affiliations:** ^1^Postgraduate Program in Nutrition, Federal University of Santa Catarina, Florianopolis, Brazil; ^2^Translational Nutritional Neuroscience Working Group, Federal University of Santa Catarina, Florianopolis, Brazil; ^3^Nutrition Graduation Course, Federal University of Santa Catarina, Florianopolis, Brazil; ^4^Department of Nutrition, Federal University of Santa Catarina, Florianopolis, Brazil; ^5^Department of Psychiatry, Queen’s University, Kingston, ON, Canada; ^6^Inpatient Psychiatric Unit, Kingston General Hospital, Kingston Health Sciences Centre, Kingston, ON, Canada; ^7^Centre for Neuroscience Studies, Queen’s University, Kingston, ON, Canada

**Keywords:** vitamin D, cholecalciferol, depressive symptoms, social isolation, pandemics, COVID-19

## Abstract

Since the COVID-19 outbreak, studies across diverse countries have strongly pointed toward the emergence of a mental health crisis, with a dramatic increase in the prevalence of depressive psychopathology and suicidal tendencies. Vitamin D deficiency has been associated with an increased risk of mental health problems as well as individual responses to stress. Studies have discussed the relationship between low serum vitamin D concentrations and depressive symptoms, suggesting that maintaining adequate concentrations of serum vitamin D seems to have a protective effect against it. Vitamin D was found to contribute to improved serotonergic neurotransmission in the experimental model of depression by regulating serotonin metabolism. The signaling of 1,25-dihydroxyvitamin D3, the active form of vitamin D, through vitamin D receptor (VDR) induces the expression of the gene of tryptophan hydroxylase 2 (TPH2), influences the expression of serotonin reuptake transporter (SERT) as well as the levels of monoamine oxidase-A (MAO-A), the enzyme responsible for serotonin catabolism. Vitamin D also presents a relevant link with chronobiological interplay, which could influence the development of depressive symptoms when unbalance between light-dark cycles occurs. In this Perspective, we discussed the significant role of vitamin D in the elevation of stress-related depressive symptoms during the COVID-19 pandemic. It is suggested that vitamin D monitoring and, when deficiency is detected, supplementation could be considered as an important healthcare measure while lockdown and social isolation procedures last during the COVID-19 pandemic.

## Graphical Abstract

Role of vitamin D in the development of depressive symptoms. The synthesis of vitamin D from sunlight is impaired by lockdown and social distance measures imposed by the governments around the world during COVID-10 pandemic. Endogenous vitamin D synthesis initiates in the skin when 7-dehydrocholesterol (7-DHC) is converted in pre-vitamin D3 and then vitamin D3 [25(OH)D3]. It is transported through blood circulation by the vitamin D binding protein (VDBP) to the liver, the kidney, and the brain, where can be converted in its the active form [1,25(OH)2D3]. In the brain, the biological effects of 1,25(OH)2D3 are largely mediated by vitamin D receptor (VDR) through genomic mechanisms, which influence several aspects of serotonin metabolism, such as increasing serotonin synthesis by induction of the tryptophan hydroxylase 2 (TPH2) gene expression; influencing the expression of serotonin reuptake transporter (SERT) and the levels of monoamine oxidase-A (MAO-A), responsible to serotonin catabolism; and indirectly may regulate the synthesis of melatonin that improve the circadian rhythm. This mechanism can be impaired during social isolation and consequent reduction of vitamin D due to low sun exposure during the pandemic, which could contribute to the development of depressive symptoms.

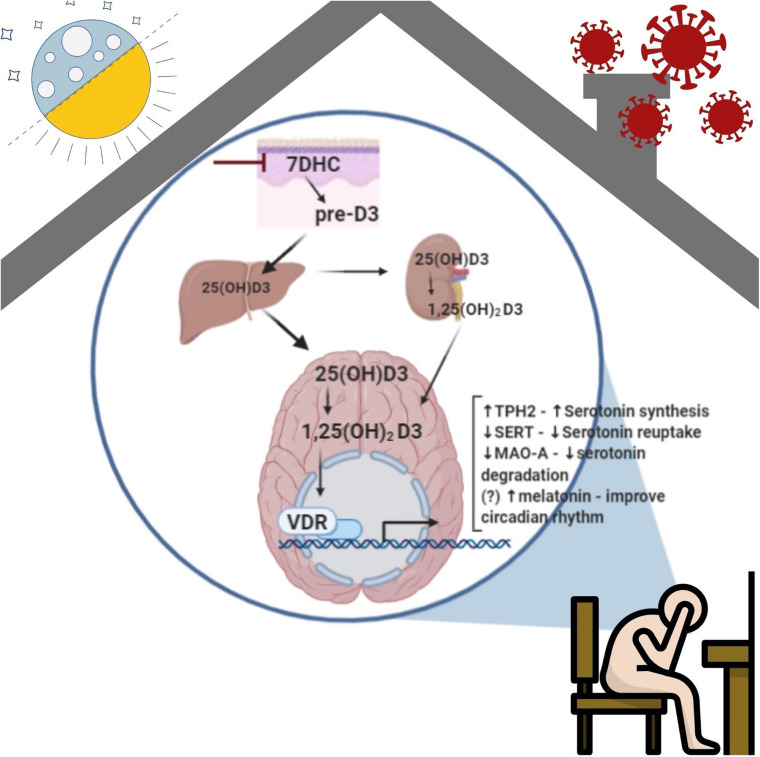

## Depression and COVID-19 Pandemic

The current pandemic of coronavirus (COVID-19) has imposed social isolation measures, forcing many people to stay confined at home, canceling routine outdoor activities (working routine, sidewalks, exercises in fitness centers, and park visits), and limiting social interaction among friends, coworkers, and family. A study concentrated on a general population from China within the first 2 weeks of the COVID-19 outbreak indicated that the lifestyle changes prompted individuals to stay indoors for over 20–24 h per day (Wang et al., 2020).

Since the COVID-19 outbreak, studies across diverse countries have strongly pointed toward the emergence of a mental health crisis, with a dramatic increase in the prevalence of depressive psychopathology and suicidal tendencies. A systematic review of the instances of mental health conditions during the period of the COVID-19 pandemic showed that the prevalence of depressive symptoms ranged from 14.6 to 48.3% across all populations (China, Spain, Italy, Iran, the United States, Turkey, Nepal, and Denmark) (Xiong et al., 2020). In the United States, the prevalence of symptoms associated with depression increased more than threefold during the COVID-19 pandemic compared to the times before the viral outbreak (8.5% before to 27.8% during COVID-19) (Ettman et al., 2020). In London, after lockdown the prevalence of feeling worse on the depression was 12.8% and to anxiety was 12.3% in people with 50 years or over (Robb et al., 2020). Restriction of public circulation and social isolation, despite those being essential measures to contain the pandemic, has been postulated to be responsible for increased stress, loneliness, and unhealthy lifestyle habits (Luo et al., 2020; Smith and Lim, 2020).

Recently, a longitudinal study involving older adults (Santini et al., 2020) demonstrated that social disconnectedness could contribute to a greater risk of depression and anxiety, which has also been reported in adolescent and adult populations (Smith and Lim, 2020). In a survey conducted by UNICEF, Australia on 1,000 young people (aged 13–17 years), almost half of the respondents asserted that COVID-19 had negatively affected their levels of stress and anxiety. An elevated level of stress, fear, irritability, frustration, and boredom was reported by individuals who were in social isolation and had experienced a major deflection from their usual routine and interaction with other people (Smith and Lim, 2020). In addition to the increase in the risk of mental health problems, the pandemic has also exacerbated preexisting psychiatric conditions. Vulnerability to psychological stress might depend on social support, isolation time, COVID-19 infection-related factors, the amount of mass media exposure, physical activity, and eating patterns (Kontoangelos et al., 2020).

## Vitamin D and Sunlight Exposure: Breakdown by Social Isolation Measures During COVID-19 Pandemic

Vitamin D deficiency has been associated with an increased risk of mental health problems as well as individual responses to stress (Amrein et al., 2020). There are several risk factors related to vitamin D deficiency such as obesity, dark skin, living in countries with low sunlight incidence, gastrointestinal malabsorption, renal insufficiency, liver disease, and the use of covered clothing and sunscreen (Kennel et al., 2010; Holick et al., 2011). Furthermore, reduced exposure to sunlight, thereby reducing the biosynthesis of vitamin D in the skin, is a strong factor in the pathophysiology of vitamin D deficiency and studies have been demonstrated that sun exposure can enhance vitamin D synthesis (Jorde et al., 2010; Chel et al., 2011; Macdonald et al., 2011; Chalcraft et al., 2020).

After adequate exposure to sunlight, the conversion of 7-dehydrocholesterol (7-DHC) to 25-hydroxycholecalciferol [25(OH)D] takes approximately 8 h and an additional time to enter the dermal capillary bed. The daily exposure of 20% of the body surface is sufficient to increase 25(OH)D, which points toward the importance of sunlight in the maintenance of vitamin D levels at the appropriate concentration (Wacker and Holick, 2013). The half-life of 25(OH)D was estimated to 15 to 25 days, approximately (Lips, 2007; Jones et al., 2014). The half-life of serum 25(OH)D was estimated to be ∼82 days when calculated after one single loading dose of 100 000 IU of cholecalciferol was administered at the beginning of the study, followed by a daily dose of 4800 IU from days 7 to 20, and none from day 21 (Oliveri et al., 2015). In Nordic countries, which experience low amounts of sunlight, vitamin D concentrations were found to be reduced by 20 to 40% during the winter (Jorde et al., 2010). These data suggest that vitamin D supplementation is crucial in individuals who experience long periods of indoor activities and/or low sunshine exposure.

Social isolation and lockdown measures caused a reduction in the time spent outdoors and possibly less exposure to sunlight necessary to maintain vitamin D concentrations. When associated with a change in eating habits, with a predominance of meals ordered through food delivery joints, which have a reduced nutritional and vitamin D content, it could also reduce the daily amount of vitamin D for organism maintenance. One study conducted in Verona (Italy) during the COVID-19 pandemic, which intends to investigate whether social isolation could impact the vitamin D status of the general population, conclude that this was not an issue in Verona (the region in which data was collected) (Lippi et al., 2021). Despite scarce publications address this issue, research groups, and professionals already start to concern about vitamin D deficiency during the COVID-19 pandemic. Some postulate that home reclusion could lead to a surge of vitamin D deficiency around the world, which has been associated with developing type 2 diabetes, cognitive decline, malignant neoplasms, autoimmune diseases, cardiovascular diseases, osteoporosis, risk of fall in the elderly, and overall mortality (Alpalhão and Filipe, 2020). In fact, the diminished vitamin D intake and sun exposure might result in severe manifestations since the worldwide prevalence of vitamin D deficiency had been documented as a health concern even before the pandemic (Luo et al., 2018; Zhou et al., 2019; Amrein et al., 2020).

## Vitamin D and Depressive Symptoms

Vitamin D low levels have been previously associated with the risk of depressive symptoms and depression worldwide (Song et al., 2016; Vidgren et al., 2018; Yao et al., 2018; Ceolin et al., 2020; Köhnke et al., 2020). However, few publications have been discussing this relationship during the COVID-19 pandemic (Di Nicola et al., 2020; Mehta et al., 2021). Mehta et al. (2021) discussed that vitamin D can be hypothesized to trigger as well as sustain the psychiatric symptoms and can also be expected to alleviate the psychiatric manifestations in COVID-19. An observational study in Rome showed that low 25-hydroxyvitamin D serum levels were significantly associated with higher psychological distress in patients with mood disorders during the COVID-19 outbreak (Di Nicola et al., 2020).

The mechanisms associated with vitamin D antidepressant effects at the cellular level are under investigation in experimental studies, and some hypotheses have been presented in different reports in the literature. The VDR and some cytochrome P450 enzymes, which are responsible for the transformation of vitamin D into its active form, were found in cerebral cells and in brain areas that have been implicated in the pathophysiology of depression (He et al., 2020). The optimal vitamin D status was found to contribute to improved serotonergic neurotransmission deficits in an experimental model of depression by regulating serotonin synthesis (Sabir et al., 2018). To increase serotonin concentrations, tryptophan must first be transported across the blood-brain barrier and metabolized by TPH2. The signaling of 1,25-dihydroxycholecalciferol [1,25(OH)2D3], the active form of vitamin D, through VDR induces the expression of the gene TPH2 (Kaneko et al., 2015; Patrick and Ames, 2015). Moreover, 1,25(OH)2D3 influences the expression of SERT as well as the levels of MAO-A, the enzyme responsible for serotonin catabolism (Sabir et al., 2018).

Another important aspect related to vitamin D and brain function is its link with chronobiological interplay. Indeed, the interface between vitamin D and the circadian system has been revealed as both plasma concentrations of 1,25(OH)2D3 and vitamin D binding protein (DBP) display circadian oscillation patterns (Jones et al., 2017). However, available evidence suggests a mediatory role of vitamin D in the sleep-wake cycle, since the lower concentrations of vitamin D were correlated with impaired sleep quality and abbreviated sleep duration (Jones et al., 2017; Muscogiuri et al., 2019). Melatonin, a hormone involved in the regulation of circadian rhythm, is synthesized from the metabolism of serotonin. The widespread presence of VDR has been documented in postmortem human brain samples, in areas involved in sleep regulation, which are also brain regions that showed the strongest expression of the enzyme 1-alpha-hydroxylase, enzymes responsible for the formation of 1,25(OH)2D3 (Eyles et al., 2005). In this way, it is possible to hypothesize that the chronic combined deficits of vitamin D and sleep-wake cycle impairment due to social isolation measures and lockdown might play an essential role as a modulator of amplified depressive symptomatology and/or the pathophysiology of major depressive disorder (MDD).

Depression has also been associated with mitochondrial dysfunctions and an increase in the formation of reactive oxygen species (ROS) inducing redox to unbalance and reduction in the energy production (adenosine triphosphate-ATP) (Caruso et al., 2019). Oxidative stress activates several transcription factors, such as nuclear factor kappa B (NF-kB) leading to the production of pro-inflammatory cytokines and other inflammatory molecules acting as potent inducers of inducible nitric oxide synthase (iNOS) (Morris and Berk, 2015). Inflammation may induce depression through many mechanisms such as an alteration in the formation of key transmitters such as serotonin and an increase in Ca^2+^ signaling (Leonard and Maes, 2012; Berridge, 2017). Also, dysfunction in mechanistic target of rapamycin (mTOR) signaling can lead to ROS production and the mTOR inhibition enhance sirtuin signaling contributing to mitochondrial integrity (Mocayar Marón et al., 2020). Vitamin D and melatonin share common signaling pathways that mediate homeostatic mitochondrial function, which includes the downregulation of mTOR, iNOS, and NF-κB pathways that are involved in increasing oxidative stress and cell damage. Also, Vitamin D and melatonin decrease oxidative stress by upregulation of sirtuin 1 (SIRT-1) and adenosine monophosphate-activated protein kinase (AMPK) pathways and activating antioxidant defense pathways by expression of antioxidant proteins. These pathways are crucial to avoid abnormal inflammatory responses related to oxidative stress and apoptosis (Mocayar Marón et al., 2020).

The studies aiming to investigate the antidepressant effect of vitamin D supplementation when depression was already diagnosed observed conflicting results (Alavi et al., 2019; Alghamdi et al., 2020; Okereke et al., 2020; Vellekkatt et al., 2020; Zhu et al., 2020). To mention some of them, in randomized clinical trials (RCT), Vellekkatt et al. (2020), Alghamdi et al. (2020), and Alavi et al. (2019) observed a reduction of depressive symptoms after vitamin D supplementation in patients with different stages of depression. Nevertheless, Okereke et al. (2020) and Zhu et al. (2020) did not observe improvements in depressive symptoms after vitamin D supplementation. Data from a previous meta-analysis with RCT showed that supplementation with vitamin D (≥800 I.U. daily dose) had a positive effect when compared to the effect of antidepressants (Spedding, 2014). But, a separate meta-analysis did not support this evidence in adults and pointed out that the variability in methodological attributes of the studies included in the meta-analysis (differences in vitamin D doses, time of intervention duration, and assessment of depression) could influence the conclusions (Gowda et al., 2015). Despite all this uncertainty about the effects of vitamin D supplementation to treat depression, when the preventive effect of adequate concentrations of vitamin D was investigated in observational studies, the results are promising. Two systematic reviews and meta-analysis with population-based epidemiological studies (Ju et al., 2013; Li et al., 2019) showed an inverse association between serum 25(OH)D and the risk of depression and, in the pooled estimation analysis, an increase of 10 ng/mL in serum concentrations of vitamin D might have a protective effect against depression. The data from these two meta-analyses leads us to postulate that the monitoring of vitamin D during long periods of indoor activities associated with reduced time to sunlight exposure is a reasonable health measure, as well as the supplementation to maintain adequate vitamin D concentrations.

## Vitamin D and COVID-19

A growing body of literature has been discussing a possible relationship between 25(OH)D and COVID-19, in which vitamin D may contribute to reducing the severity of illness caused by COVID-19 (Bergman, 2020; Jakovac, 2020; Marik et al., 2020; Mitchell, 2020; Panarese and Shahini, 2020). The main discussion is that Vitamin D could exert protective effects against respiratory infections, due to its immunomodulatory and anti-inflammatory roles, which have been highlighted in patients with community-acquired infections and acute respiratory failure (Zhou et al., 2019; Bergman, 2020; Mitchell, 2020; Panarese and Shahini, 2020). COVID-19 has been shown to affect the neuroendocrine-immune system wherein it suppresses its activity. The neuroendocrine-immune system has been implicated in stress response and coping strategies (Nami et al., 2020).

One study performed *in vitro* showed that the active form of Vitamin D, calcitriol, exhibits significant potent activity against SARS-CoV-2 in African green monkey kidney cells, human hepatoma cells, and human nasal epithelial cells (Mok et al., 2020). In fact, the interest in the field has been growing and some systematic reviews and meta-analysis were performed to measure the risk of infection and severity of COVID-19 due to low levels of 25(OH)D in observational studies (Pereira et al., 2020; Kazemi et al., 2021; Liu et al., 2021; Petrelli et al., 2021; Teshome et al., 2021). Pereira et al. (2020) reported that severe cases of COVID-19 present 64% (OR = 1.64; 95% CI = 1.30–2.09) of vitamin D deficiency and that insufficiency increased hospitalization (OR = 1.81;95% CI = 1.41–2.21) and mortality (OR = 1.82; 95% CI = 1.06–2.58). Kazemi et al. (2021) found that vitamin D deficiency was a higher risk of SARS-CoV-2 infection (OR = 1.77; 95% CI = 1.24–2.53) and presented a higher severity (OR = 2.57; 95% CI = 1.65–4.01). Liu et al. (2021) showed that vitamin D deficiency or insufficiency also was associated with an increased risk of COVID-19 (OR = 1.43; 95% CI = 1.00–2.05). Petrelli et al. (2021) revealed that deficient vitamin D values showed a higher risk of COVID-19 infection (OR = 1.26; 95% CI = 1.19–1.34) and worse severity (OR = 2.6; 95% CI = 1.84–3.67) and higher mortality (OR = 1.22; 95% CI, 1.04–1.43). Teshome et al. (2021) found that vitamin D deficiency was 80% more likely to acquire COVID-19 infection (OR = 1.80; 95%CI = 1.72–1.88).

Concerning clinical trials, only one systematic review and metanalysis were performed to identify the effect of vitamin D on COVID-19, and 32 clinical protocol was identified but only three studies have published results to-date and the results are controversial (Bassatne et al., 2021). In fact, there are some challenges in vitamin D research and some authors have provided recommendations for the design of randomized clinical trials of vitamin D supplementation to prevent COVID-19 and to provide evidence-based guidance for clinicians and public health leaders (Camargo and Martineau, 2020).

## Conclusion

In the light of these evidence, it is possible to postulate that vitamin D deficiency could play a significant role in the elevation of stress-related depressive symptoms during the COVID-19 pandemic. It has been postulated that vitamin D is involved in the serotoninergic system, influencing serotonin metabolism and contributing to circadian rhythm maintenance, which are important aspects of the development of depressive symptoms. In order to reduce physical and mental health risks associated with vitamin D deficiency, it is suggested that vitamin D monitoring and, when deficiency is detected, supplementation could be considered as an important healthcare measure while lockdown and restrictive social isolation procedures last during the COVID-19 pandemic.

## Data Availability Statement

The original contributions presented in the study are included in the article/supplementary material, further inquiries can be directed to the corresponding author.

## Author Contributions

All authors listed have made a substantial, direct and intellectual contribution to the work, and approved it for publication.

## Conflict of Interest

The authors declare that the research was conducted in the absence of any commercial or financial relationships that could be construed as a potential conflict of interest.
